# Computational Insights Into the Electronic Structure and Magnetic Properties of Rhombohedral Type Half-Metal GdMnO_3_ With Multiple Dirac-Like Band Crossings

**DOI:** 10.3389/fchem.2020.00558

**Published:** 2020-07-17

**Authors:** Yu Chang, Sung-Ryong Moon, Xin Wang, Rabah Khenata, H. Khachai, Minquan Kuang

**Affiliations:** ^1^Tonghua Normal University, Tonghua, China; ^2^Department of Electronic Engineering, Wonkwang University, Iksan, South Korea; ^3^Laboratoire de Physique Quantique de La Matiere et de Modelisation Mathematique (LPQ3M), Université de Mascara, Mascara, Algeria; ^4^Laboratoire D'etude des Materiaux & Instrumentations Optiques, Physics Department, Djillali Liabès University of Sidi Bel-Abbès, Sidi Bel Abbès, Algeria; ^5^School of Physical Science and Technology, Southwest University, Chongqing, China

**Keywords:** half-metal, spintronics, DFT, electronic structures, rhombohedral, spin-polarization

## Abstract

In spintronics, half-metallic materials (HMMs) with Dirac-like cones exhibit interesting physical properties such as massless Dirac fermions and full spin polarization. We combined first-principles calculations with the quasi-harmonic Debye model, and we proposed that the rhombohedral GdMnO_3_ is an HMM with multiple linear band crossings. The physical properties of GdMnO_3_ were studied thoroughly. Moreover, the changes of multiple linear band crossings and 100% spin polarization under spin-orbit coupling as well as the electron and hole doping were also investigated. It is noted that such spin-polarized HMMs with linear band crossings are still very rare in two-dimensional and three-dimensional materials.

Different from traditional electronics, spintronics mainly use spin rather than charge as a carrier for transmitting and processing information (Awschalom and Flatté, 2007), greatly improving the performance and effectively reducing the power consumption of electronic devices. The field of spintronics has developed rapidly in recent years, but it still faces many challenges, such as long distance spin transport, spin-polarized carrier generation and injection, and spin manipulation and detection. In response to these problems, scientists have proposed a series of spintronic materials such as spin gapless semiconductors (SGS) (Wang, 2008, 2017; Wang et al., 2010, 2016, 2017, 2018; Han et al., 2018), zero-gap half-metals (HMs) (Du et al., 2013), Dirac HMs (Ishizuka and Motome, 2012; He et al., 2016; Liu et al., 2017; Zhang et al., 2017), dilute magnetic semiconductors (DMS) (Furdyna, 1988), and bipolar Magnetic Semiconductor (BMS) (Li et al., 2012).

Currently, the new generation of spintronics also faces a problem of achieving zero-energy consumption and ultra-high speed for electronic transmission. Dirac-type half-metallic materials are excellent candidates for achieving these goals because they have the advantages of having massless Dirac fermions and high spin polarization. The first Dirac half-metal (DHMs) was theoretically verified in triangular ferromagnet (Ishizuka and Motome, 2012). Since then, research on DHMs has mainly focused on two-dimensional materials (He et al., 2016; Liu et al., 2017). Very recently, a three-dimensional material, MnF_3_ with multiple Dirac cones near the Fermi level was predicted by Jiao et al. (2017). Compared to the materials with a single-Dirac-cone DHM, materials with multiple Dirac band dispersions can exhibit higher carrier transmission efficiency and stronger non-linear electromagnetic response. Subsequently, LaMnO_3_ (Ma et al., 2018) with the $R\bar{3}c$ type structure was proposed by Du's group in 2018 to display complete spin-polarization and multiple Dirac cones around the Fermi level.

Unfortunately, the types and number of DHMs is extremely limited. Therefore, it is necessary to design more HMMs with Dirac-like band crossings and 100% spin polarization. In this work, we will study the electronic structures, magnetism, and thermodynamic properties of the $R\bar{3}c$ type GdMnO_3_ using first-principles calculations incorporating the quasi-harmonic Debye model (QDM). Furthermore, the spin-orbit coupling, and the electron and hole doping effects were taken into consideration to examine the stability of the electronic structure. A more detailed description of the Computational Methods is provided in the Supplementary Information.

The $R\bar{3}c$*-*type GdMnO_3_ crystal structure with hexagonal setting is shown in Figure 1 (Left). The GdMnO_3_ crystal structure belongs to space group number 167 and the optimized equilibrium lattice parameters for the FM state are a = b = 5.63 Å and c = 13.17 Å. The six Mn atoms have four types of magnetic orderings, namely, FM, NM, AFM-1, and AFM-*2*. As shown in Figure 1 (Right), the spin orderings of the six Mn are ↑↑↑↑↑↑, ↑↓↑↓↑↓, and ↑↑↑↓↓↓ respectively, for FM, AFM-1 and AFM-*2* configurations. By fully relaxing the crystal structures of GdMnO_3_ and checking the magnetic structures of this GdMnO_3_. From Figure 1 (Right), one can conclude that the most stable magnetic phase is the FM phase due to its lowest total energy. The calculated total magnetic moment of this material under FM and equilibrium lattice constant is 24 μ_B_ per unit cell, and arises mainly from the Mn atoms (the spin density of GdMnO_3_ is given in Figure 2), with the atomic magnetic moment of each Mn atom of ~4.4 μ_B_.

**Figure 1:**
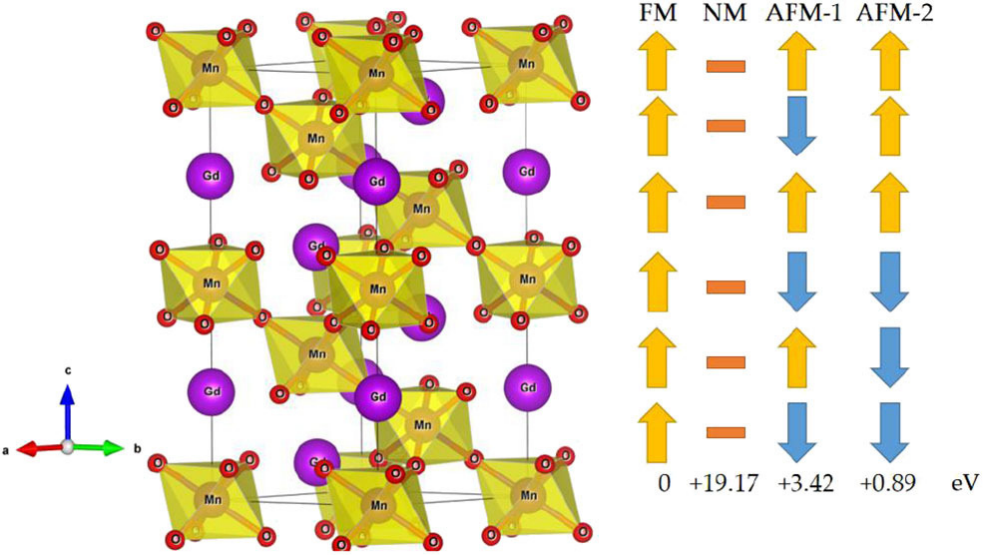
**(Left)** Crystal structures of the $R\bar{3}c$-type GdMnO_3_ material with hexagonal structure; **(Right)** four magnetic structures, i.e., FM, NM, and two AFM structures (labeled as AFM-1, and AFM-2).

**Figure 2:**
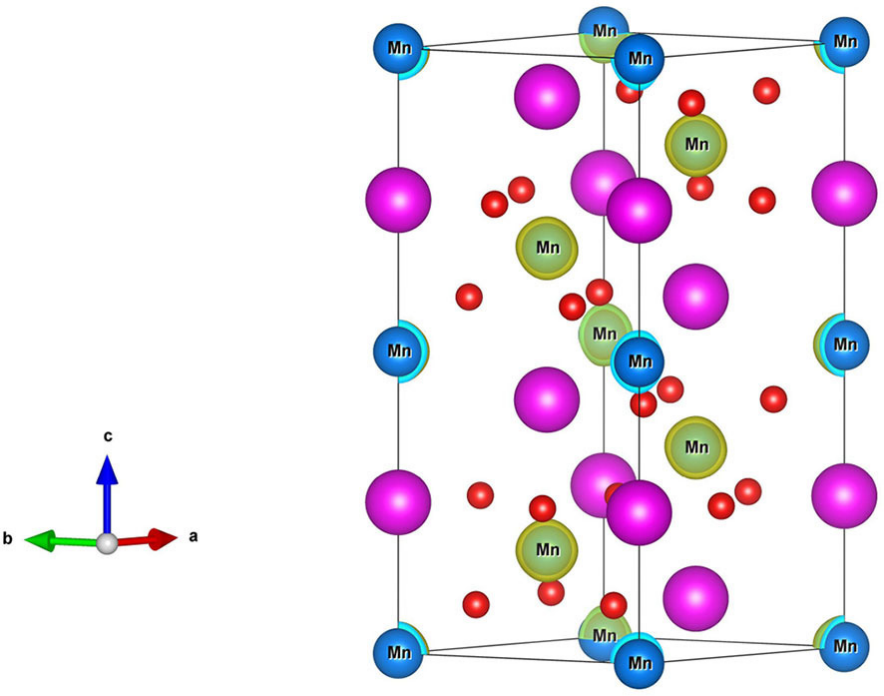
Spin-density of GdMnO_3_ obtained by the DFT+U method.

Figure 3 shows the spin-polarized band structures of GdMnO_3_ obtained by DFT+U calculations. To accurately describe the electronic structures of GdMnO_3_, the on-site Coulomb interaction U and exchange interaction J were set to 10.1 and 0.88 eV, respectively, for Mn-3*d* based on reference (Ma et al., 2018). An examination of the band structure shows that while the band gap (~4.73 eV) and the half-metallic band gap (~0.75 eV) is found in the spin-down channel, the bands in the spin-up channel show metallic properties, suggesting that GdMnO_3_ is a half-metallic material (Anjami et al., 2017; Jiao et al., 2017; Ma et al., 2018). Normally, a large energy gap (difference between the energies of the highest occupied and lowest unoccupied in the spin-down channel) and a half-metallic band gap (difference between the energies of highest occupied and the Fermi level in the spin-down channel) are considered to be evidence of robust half-metallic behaviors.

**Figure 3:**
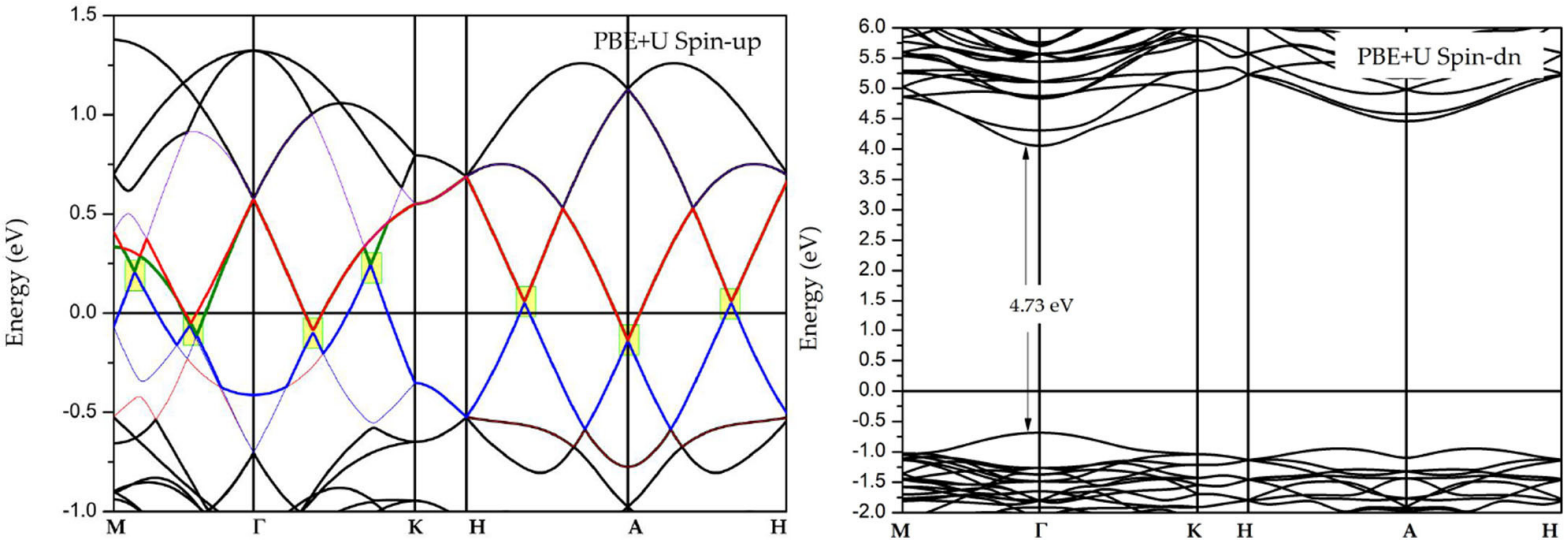
Spin-polarized band structure of GdMnO_3_ obtained by the DFT+U method.

Interestingly, in the spin-up channel, there are some Dirac-like band crossings that are very close to the Fermi level. In Figure 3, we used yellow squares to highlight the regions of the Dirac-like crossing points near the Fermi level. Clearly, three Dirac-like crossing points are present along the M–Γ path, two Dirac-like crossing points are present along the Γ-K path, one Dirac-like crossing point is located at the A high symmetry point, and a Dirac-like crossing point is found along the A–H path.

Figure 4 shows the TDOS and PDOS of GdMnO_3_ in both spin directions obtained using the DFT+U method. Spin polarization (%) is a highly important parameter and can be expressed as Jiao et al. (2017): P $=\frac{\left|N↑\left(E_{f}\right)-N↓\left(E_{f}\right)\right|}{\left|N↑\left(E_{f}\right)+N↓\left(E_{f}\right)\right|}\times 100$ where *N* ↑(*E*_*f*_) and *N* ↓ (*E*_*f*_). Here, *N* ↑ (*E*_*f*_) is the number of the spin-up states and *N* ↑ (*E*_*f*_) is the number of the spin-down states. An examination of Figure 4A shows that the *P*-value of GdMnO_3_ is 100%, indicating that GdMnO_3_ can be selected for using in future spintronic devices.

**Figure 4:**
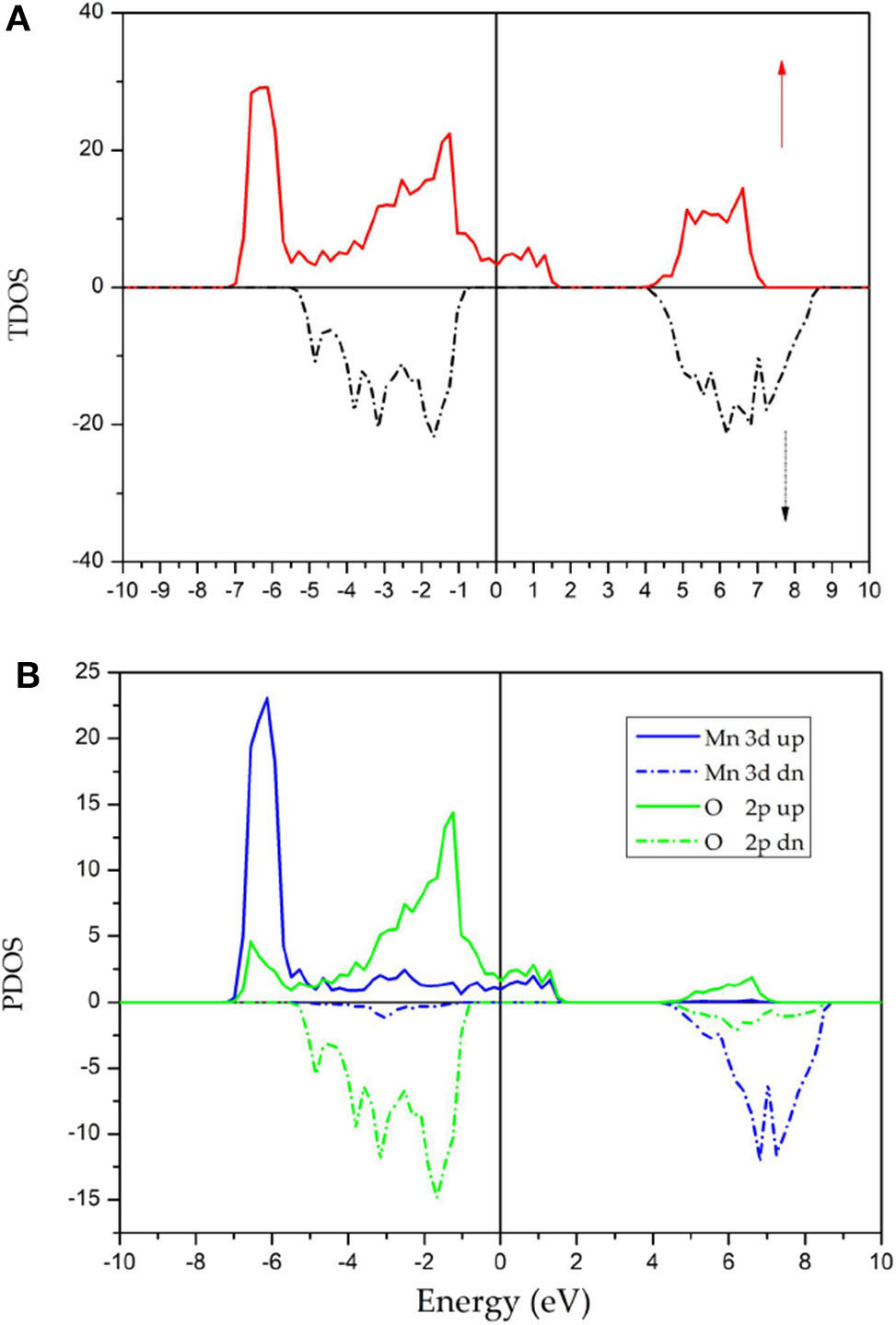
Calculated TDOS **(A)** and PDOS **(B)** of GdMnO_3_ obtained using the DFT+U method.

An examination of Figure 4B shows a clear energy gap in the vicinity of the Fermi level in the spin-down channel; however, the spin-up electrons show metallic behavior. For the spin-up channel, the density of states primarily arises from the *d* states of the Mn atoms and the *p* states of O atoms. It is important to note that the DOS peak at approximately −7 eV (7 eV) of the spin-down channel (spin-up channel) is induced due to the strong spin splitting (Qin et al., 2017; Han et al., 2019) of the Mn atoms. At the same time, the nearly symmetrical DOS of O in both spin directions suggest that the atomic magnetic moments of O can be ignored.

Because the GdMnO_3_ system contains heavy atoms, the effect of the spin-orbit coupling (SOC) should be discussed in this work. Figure 5 shows the band structures of GdMnO_3_ obtained by DFT+U+SOC calculations. It is observed that the Dirac-like band crossings is still present when the SOC is taken into account, namely, the conduction and valence bands are still degenerate. Due to the robustness with respect to the SOC effect, GdMnO_3_ features long spin coherence which is helpful for spintronic applications.

**Figure 5:**
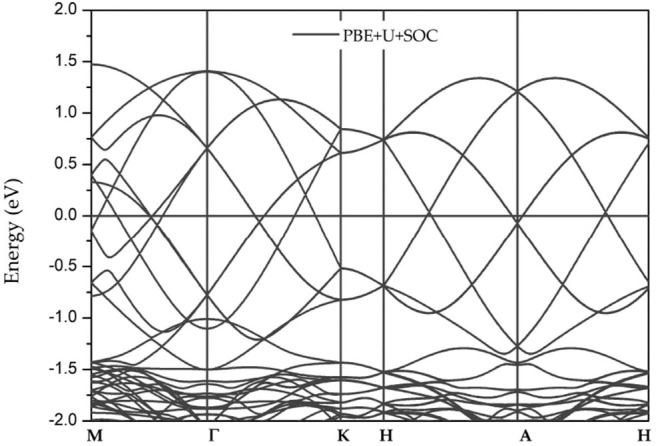
Band structures of GdMnO_3_ obtained using the DFT+U+SOC method.

It is well-known that electron and hole doping may influence the stability of the electronic structure around the Fermi level. Therefore, in this work, we will study the effect of electron and hole doping on the band structure. The doping concentration of each atom is 0.025 carrier, and the obtained band structures under the electron doping and under hole doping are shown in Figures 6A–**D**, respectively. An examination of these figures shows that electron doping will increase the energy of the Fermi level, while hole doping will decrease the Fermi level energy. Both electron and hole doping have no obvious effect on the Dirac-like band crossings and half-metallic states (see Figures 6A,C) with the exception of changing the location of the Dirac-like band crossing points. The carrier concentration, can be adjusted by use of a gate voltage (Thelander et al., 2004).

**Figure 6:**
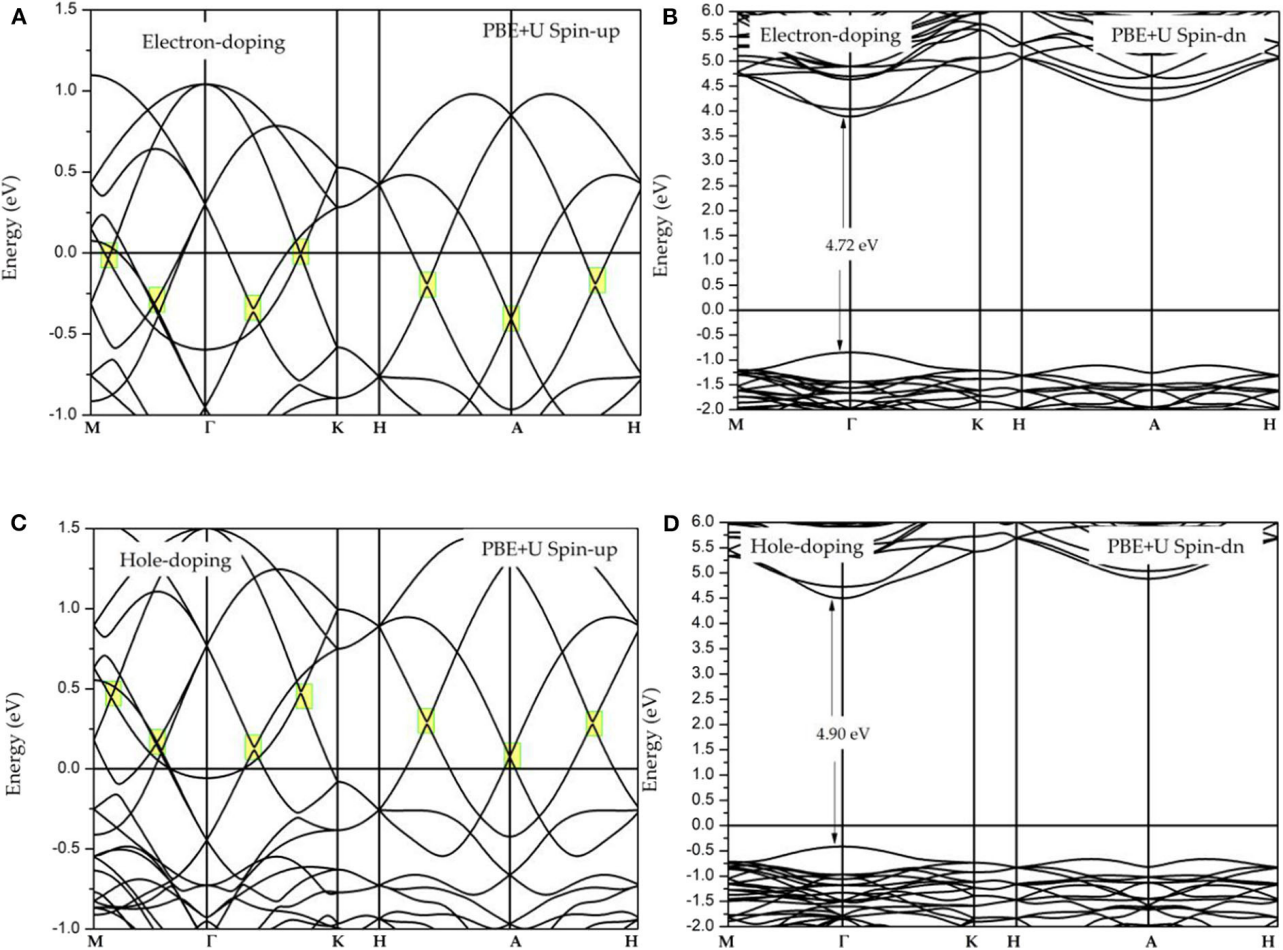
**(A,B)** Spin-polarized band structures of GdMnO_3_ under electron doping; **(C,D)** spin-polarized band structures of GdMnO_3_ under hole doping.

Then, we calculated some thermodynamic parameters to understand the thermodynamic behavior of GdMnO_3_ under high temperature and high pressure limits. As mentioned in the Computational method section, in this work, QDM is used to investigate the thermodynamic properties, and the investigated temperature and pressure regions were 0–2,000 K and 0–50 GPa, respectively.

Figure 7 shows the relationship between the *V*/*V*_0_ rate of change and temperature and pressure, where *V*_0_ is the initial volume and *V* is the final volume for a given pressure. In Figure 7 (Left), we can clearly find that for a given fixed temperature, the rate *V*/*V*_0_ decreases with increasing pressure. This is a negative correlation; in other words, the slope is negative. As observed from Figure 7 (Right), the *V*/*V*_0_ rate and temperature are positively correlated.

**Figure 7:**
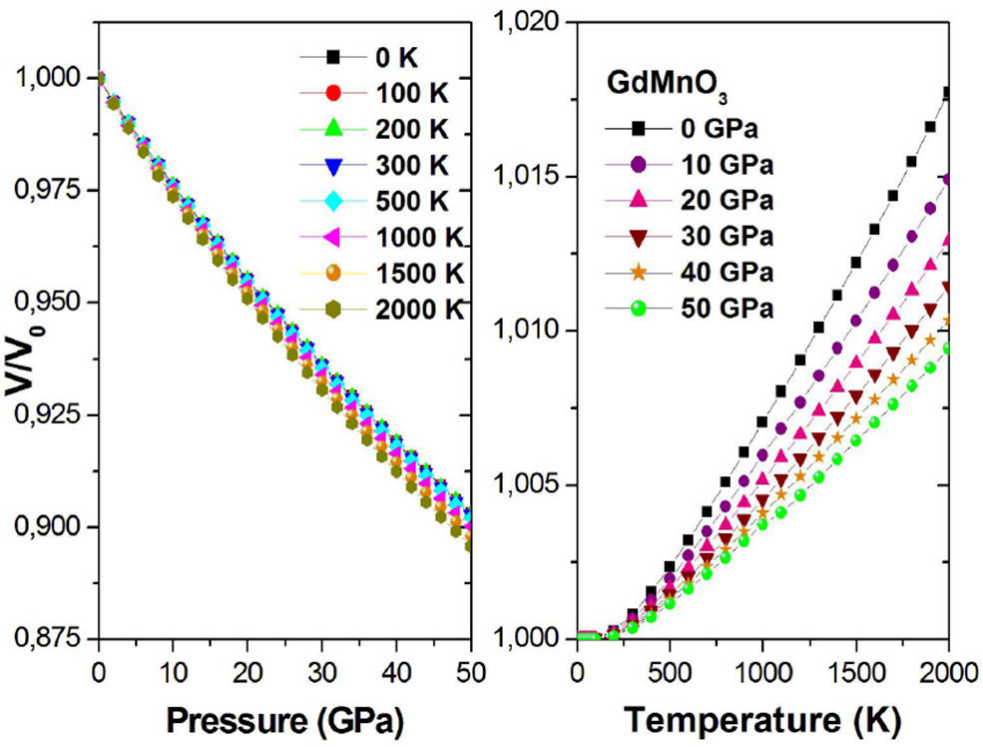
*V*/*V*_0_ ratio as a function of the pressure (temperature) at different given temperatures (pressures).

The relationships between α_*V*_ on the one hand and pressure and temperature on the other hand are shown in Figure 8. For a fixed pressure, with increasing temperature, α_*V*_ increases first sharply and then slowly. This may be due to the higher pressure limiting the rise of α_*V*_. Figure 8 (Right) shows that for a constant temperature, although the increase in the pressure will gradually decrease α_*V*_, for higher temperature, the increase in the pressure will only increase the initial value of α_*V*_ without affecting the trend.

**Figure 8:**
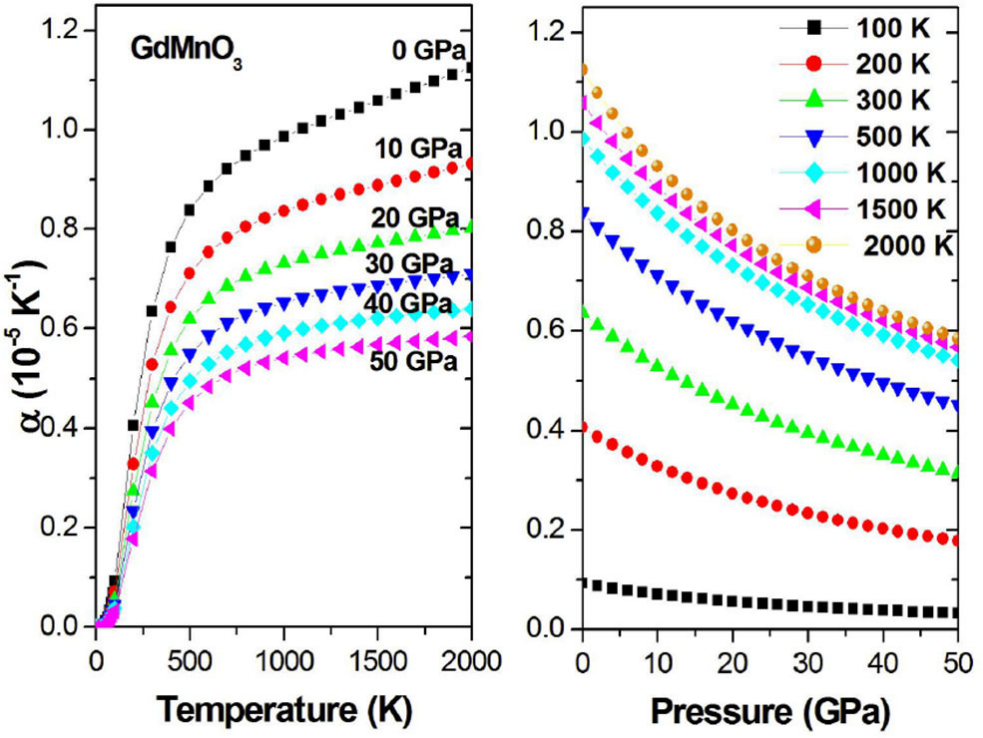
α_*V*_ as a function of the temperature (pressure) at different given pressures (temperatures).

Using the Grüneisen parameter (γ) to predict the thermodynamic properties of the material at high temperatures and pressures, we can evaluate the effect of the crystal anharmonicity. Figure 9 shows the relationship between γ on the one hand and the pressure and temperature on the other hand, and it is observed that with increasing temperature or decreasing pressure, the pressure/temperature relationship increases accordingly.

**Figure 9:**
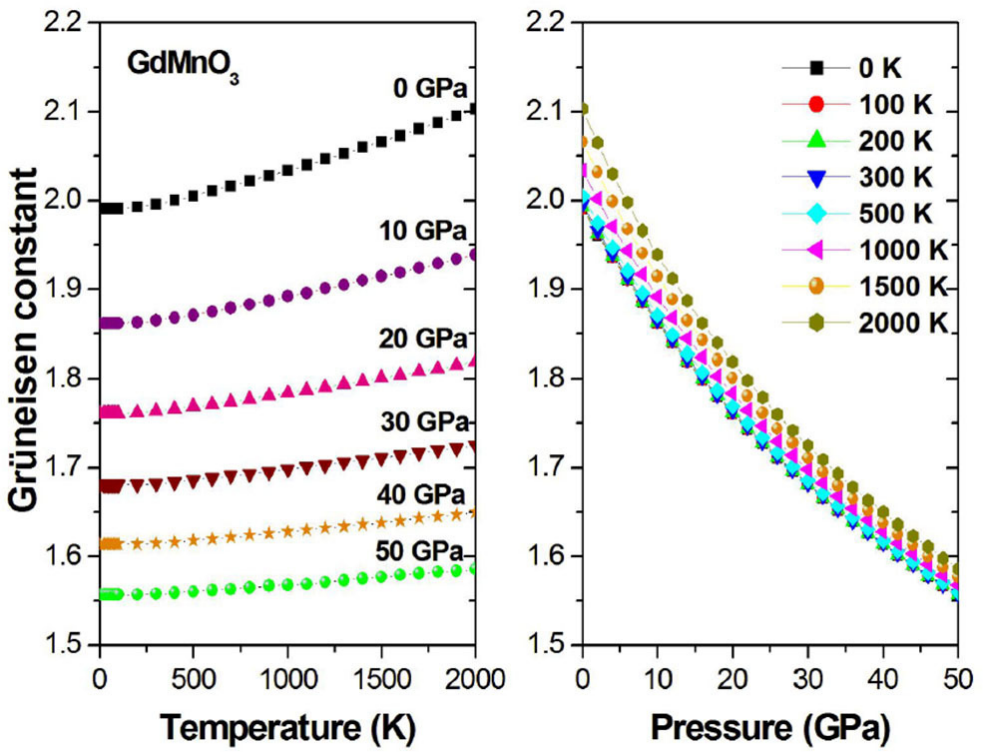
Grüneisen constant γ as a function of the temperature (pressure) at different given pressures (temperatures).

Figure 10 shows that the heat capacity of GdMnO_3_ shows a close to cubic dependence on the temperature (T^3^) at low temperatures, suggesting that lattice vibrations will increase with increasing temperature. Moreover, the high temperature value is constant, as stated by the Dulong-Petti limit, indicating that the thermal energy at high temperature excites all phonon modes. The increase in the absolute pressure increase is a negative effect and will stop lattice vibrations.

**Figure 10:**
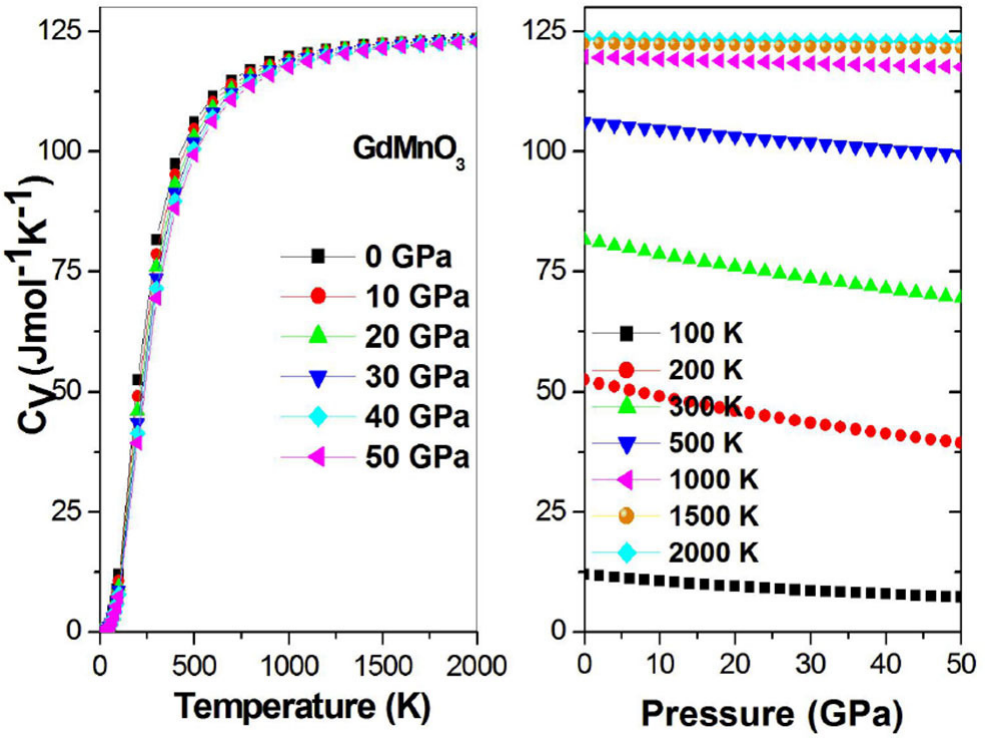
Heat capacity *C*_*V*_ as a function of the temperature (pressure) at different given pressures (temperatures).

Figure 11 shows the Debye temperature and its relation to the temperature at fixed pressures and the Debye temperature and its relationship to the pressure at fixed temperatures. The value of θ_*p*_ has a negative quasi-linear relationship with temperature and a positive quasi-linear relationship with pressure.

**Figure 11:**
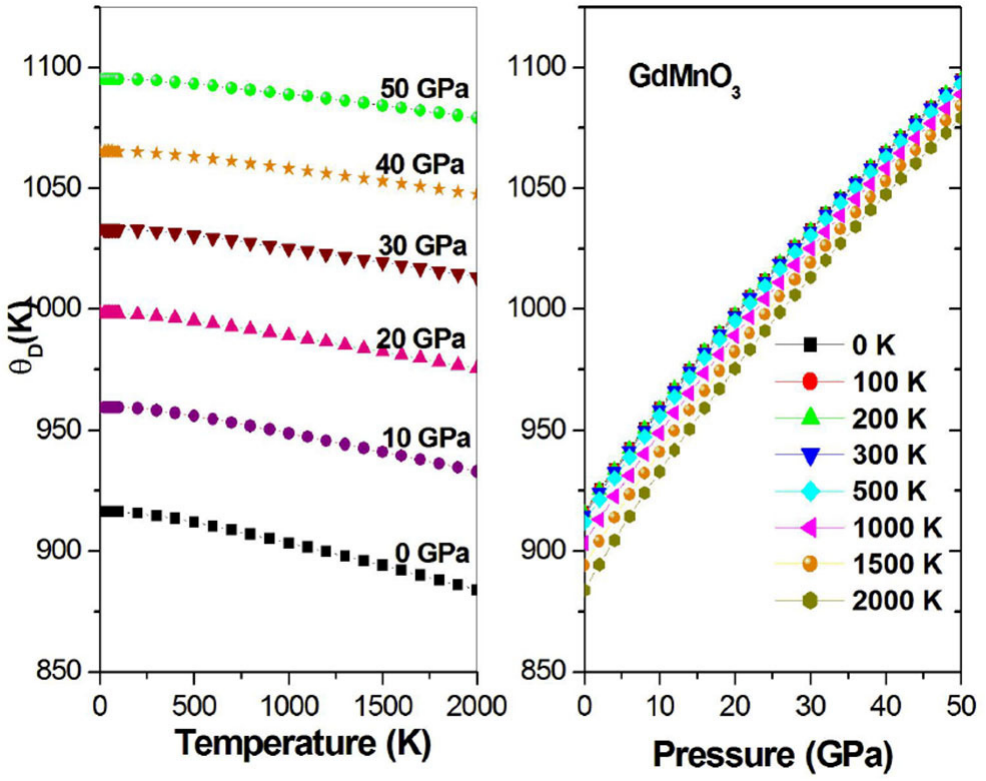
Debye temperature θ_*p*_ as a function of the temperature (pressure) at different given pressures (temperatures).

In this study, a new material, perovskite $R\bar{3}c$ type GdMnO_3_, with half-metallic behavior and multiple linear band crossing was examined and its electronic, magnetic, and thermodynamic properties were investigated via first-principles and QDM calculations. In this work, different magnetic structures were considered, namely, FM, NM, AFM-1, and AFM-2. We found that FM is the most stable due to its lowest total energy. The SOC effect has a weak effect on the electronic structure and therefore this material may have the potential for long spin coherence for long-range spin transport. The effects of electron and hole doping were also included in this calculation. We found the properties of half-metallicity and multiple linear band crossings are highly robust against electron and hole doping.

## Data Availability Statement

The raw data supporting the conclusions of this article will be made available by the authors, without undue reservation.

## Author Contributions

YC: software, methodology, and writing. S-RM: supervisor. XW, RK, and HK: reviewing and editing. MK: conceptualization. All authors contributed to the article and approved the submitted version.

## Conflict of Interest

The authors declare that the research was conducted in the absence of any commercial or financial relationships that could be construed as a potential conflict of interest.
