# Supplementary material for: Computational Insights Into the Electronic Structure and Magnetic Properties of Rhombohedral Type Half-Metal GdMnO3 With Multiple Dirac-Like Band Crossings
Source: Front Chem. 2020 Jul 17;8:558. doi: 10.3389/fchem.2020.00558 (PMC7386256; doi:10.3389/fchem.2020.00558)
Supplement: Supplementary file 1 [file Table_1.doc]

**Computational Methods**

We calculated the electronic structure and magnetic properties of the material using density functional theory as implemented VASP [1]. The exchange-correlation functional was described according to the Perdew-Burke-Ernzerhof (PBE) [2] parameterization of the GGA. To represent the interaction between the ions cores and valence electrons, we chose the projector augmented wave (PAW) method [3]. The cutoff energy of the plane wave set was set to 500 eV. We optimized the unit cell until the force and total energy were less than 0.005 eV/Å and 10^-6^ eV, respectively. In this work, the f-electrons of the Gd atom are frozen into the core, and the corresponding p and s states are included as valence electrons. Quasi-harmonic Debye model (QDM) can be used to calculate the thermodynamic properties of GdMnO_3_ [4]. In the QDM approach, the solid non-equilibrium Gibbs free energy is represented as:

$G^{*}\left(V;P,T\right)=E\left(V\right)+PV+A_{vib}\left(\theta_{D}\left(V\right);T\right)$.

Here, the total energy of the unit cell is $E\left(V\right)$, $PV$ represents the constant hydrostatic pressure condition, $A_{vib}$ is the vibrational Helmholtz free energy, and $\theta_{D}\left(V\right)$ represents the Debye temperature.

The specific form of $A_{vib}$ is given by:

$$A_{vib}\left(\theta_{D}\left(V\right);T\right)=nkT\left[\frac{9\theta_{D}}{8T}+3\ln\left(1-e^{-\frac{\theta_{D}}{T}}\right)-D\left(\frac{\theta_{D}}{T}\right)\right]$$

where $n$ is the number of the atoms per formula unit and the Debye integral $D\left(y\right)$ is given by:

$$D\left(y\right)=\frac{3}{y^{3}}\int_{0}^{y} \frac{x^{3}}{e^{x}-1}dx$$

Additionally, the Debye temperature of an isotropic solid is given by:

where $M$ represents the molecular mass per formula unit and $K_{S}$ denotes the static bulk modulus defined as:

$$K_{S}\approx K_{Static}=V\left[\frac{d^{2}E\left(V\right)}{dV^{2}}\right]$$

and $f\left(\sigma\right)$ is given by:

$$f\left(\sigma\right)=\left\{3\left[2\left(\frac{2\left(1+\sigma\right)}{3\left(1-2\sigma\right)}\right)^{2/3}+\left(\frac{1+\sigma}{3\left(1-\sigma\right)}\right)^{2/3}\right]^{-1}\right\}^{1/3}$$

Here, $\sigma$ is the Poisson ratio.

The equilibrium volume $V\left(T,P\right)$ curve (equation of states (EOS)) is obtained from:

$$\left[\frac{\partial G^{*}\left(V;P,T\right)}{\partial V}\right]_{P,T}=0$$

The isothermal bulk modulus $B_{T}$ is defined as:

$$B_{T}\left(T,P\right)=-V\left(\frac{\partial P}{\partial V}\right)_{T}$$

and is calculated at the equilibrium volume for a given $T,P$.

The heat capacity $C_{V}$ can be computed according to:

$$C_{V,vib}=3nk_{B}\left[4D\left(\frac{\theta_{D}}{T}\right)-\frac{3\theta_{D}/T}{e^{\theta_{D}/T}-1}\right]$$

The Grüneisen parameter $\gamma_{th}$ can be given as:

$$\gamma_{th}=-\frac{d\ln\theta_{D}\left(V\right)}{d\ln V}$$

based on $\gamma_{th}$, and the coefficient of thermal volume-expansion $\alpha_{V}$ is given by:

$$\alpha_{V}=\frac{\gamma_{th}C_{V,vib}}{B_{T}V}$$

References

1. Hafner J. Ab‐initio simulations of materials using VASP: Density‐functional theory and beyond. Journal of Computational Chemistry, 2008, 29(13): 2044-2078.
2. Ernzerhof M, Scuseria G E. Assessment of the Perdew–Burke–Ernzerhof exchange-correlation functional. The Journal of Chemical Physics, 1999, 110(11): 5029-5036.
3. Blöchl P E. Projector augmented-wave method. Physical Review B, 1994, 50(24): 17953.
4. Blanco M A, Francisco E, Luana V. GIBBS: isothermal-isobaric thermodynamics of solids from energy curves using a quasi-harmonic Debye model. Computer Physics Communications, 2004, 158(1): 57-72.
